# Supplementary material for: A uniform data processing pipeline enables harmonized nanoparticle protein corona analysis across proteomics core facilities
Source: Nat Commun. 2024 Jan 6;15:342. doi: 10.1038/s41467-023-44678-x (PMC10771434; doi:10.1038/s41467-023-44678-x)
Supplement: Supplementary file 1 — Supplementary Information [file 41467_2023_44678_MOESM1_ESM.pdf]

Supplementary Information

A uniform data processing pipeline enables harmonized nanoparticle protein corona analysis across proteomics core facilities

Hassan Gharibi[1#], Ali Akbar Ashkarran[2#], Maryam Jafari[3], Elizabeth Voke[4], Markita P. Landry[4,5,6,7], Amir Ata Saei[8,9]* and Morteza Mahmoudi[2]*

[1]Division of Chemistry I, Department of Medical Biochemistry and Biophysics, Karolinska Institutet, Stockholm, Sweden

[2]Department of Radiology and Precision Health Program, Michigan State University, East Lansing, MI, USA

[3]Division of ENT Diseases, Department of Clinical Science, Intervention and Technology, Karolinska Institutet, Stockholm, Sweden

[4]Department of Chemical and Biomolecular Engineering, University of California, Berkeley, Berkeley, CA, USA

[5]Innovative Genomics Institute, Berkeley, CA, USA

[6]California Institute for Quantitative Biosciences, University of California, Berkeley, Berkeley, CA, USA

[7]Chan Zuckerberg Biohub, San Francisco, CA, USA

[8]Centre for Translational Microbiome Research, Department of Microbiology, Tumor and Cell Biology, Karolinska Institutet, Stockholm 17165, Sweden

[9]Biozentrum, University of Basel, 4056 Basel, Switzerland

[#]equal contribution

*Corresponding authors: (A.A.S.) amir.saei@unibas.ch; (M.M.) mahmou22@msu.edu

Supplementary Table 1. Comparison of the protocols, LC and MS systems, method durations, and other important parameters between all the cores (with permission from ref [1]).

Core	Digestion	Digestion mode	LC system	MS system	Gradient duration	Search engine	Database	Variable modifications and other search parameters	FDR	Reduction and alkylation
1	Trypsin overnight at 37 °C	On-bead digestion	Thermo Dionex Ultimate 3000RSLCnano	Fusion Lumos	NR	MSFragger	SwissProt KB	NR*	<1% at protein and peptide levels	No
2	Trypsin overnight at 37 °C	On-bead digestion	Thermo Dionex Ultimate 3000RSLCnano	Eclipse	60 min	Proteome Discoverer 2.4	UniProt	M oxidation, acetylation and M loss on protein N-termini	<1% for high confidence and <5% for medium confidence	No
3	Trypsin overnight at 37 °C	In-solution digestion	Thermo Dionex Ultimate 3000RSLCnano	Fusion	90 min	Proteome Discoverer 2.4	NCBI	M oxidation and N/Q deamidation; acetylation, M loss and M loss plus acetylation of protein N-termini	<1% at the peptide level	Yes
4	Trypsin overnight at 37 °C	On-bead digestion	NR	Velos Elite	NR	NR	NR	NR	Between 1-2% at peptide level	No
5	Trypsin overnight at 37 °C	On-bead digestion	NR	Fusion Lumos	85 min	Protein Discoverer v.2.4	UniProt	M oxidation, N/Q deamidation, STY phosphorylation and acetylation of protein N-termini, up to 3 missed cleavages were allowed.	<5% at protein and peptide levels	No
6	Trypsin according to SOP	On-bead digestion	Bruker nanoElute system	Bruker timsTOF-PRO	55 min	PEAKS-XPro server	UniProt	M oxidation and N/Q deamidation, semi-specific search	<10% at the peptide level	Yes
7	Promega trypsin or chymotrypsin overnight at 37 °C	On-bead digestion	Waters NanoAcquity	Elite	73.5 min	PEAKS Studio 10plus	NCBI	NR	<0.5% at protein and peptide levels	Yes
8	Trypsin/Lys-C overnight	On-bead digestion	Waters NanoAcquity	Q Exactive HF-X	Total gradient of 80 min	Byonic v4.2.4	UniProt	M, H, and W oxidation, dioxidation on M and W, N/Q deamidation, and acetylation on protein N-termini, semi-specific search	<1% at the protein level	Yes, with acrylamide
9	Pierce Trypsin/Lys-C Protease mixture overnight at 37 °C	In-solution digestion	Thermo Dionex Ultimate 3000RSLCnano	Fusion Lumos	60 min	Proteome Discoverer 2.4	SwissProt	M oxidation and acetylation of protein N-termini	<1% at the peptide level; <1% for high confidence and <5% for medium confidence at protein level	Yes
10	Trypsin overnight at 37 °C	On-bead digestion	Thermo Dionex Ultimate 3000RSLCnano	Fusion Lumos	90 min	Peaks Studio 8.5	NR	NR	NR	Yes
11	Trypsin overnight at 37 °C	In-gel digestion	NR	Eclipse	NR	Proteome Discoverer 2.4	UniProt	M oxidation and N/Q deamidation	<1% at the peptide level	Yes
12	Trypsin for 1 h at 47 °C followed by 3 hours at 37 °C	On-bead digestion	Thermo Easy-nLC 1000	Fusion	NR	Proteome Discoverer 2.4	UniProt	M oxidation and N/Q deamidation	<1%	Yes
13	Trypsin for 30 min at 55 °C	On-bead digestion	Thermo Dionex Ultimate 3000RSLCnano	Fusion	90 min	Mascot 2.8	Swissprot	M oxidation	NR	No
14	Trypsin/lys C rapid digestion kit from Promega for 70 °C for 1 h	On-bead digestion	Thermo Dionex Ultimate 3000RSLCnano	Q Exactive HF	120 min	Proteome Discoverer 2.4.0.305	UniProt	M oxidation and N/Q deamidation; acetylation, M loss and M loss plus acetylation on protein N-termini	<5% at the peptide level and 1% at the protein level	Yes
15	Trypsin/Lys-C mixture using the manufacturer protocol	On-bead digestion	Thermo Thermo Dionex Ultimate 3000RSLCnano	Eclipse	100 min	Proteome Discoverer 2.2.0.388	UniProt	M oxidation, acetylation of the N-terminus and S phosphorylation	<5% at the peptide and protein levels	Yes
16	Trypsin overnight at 37 °C	On-bead digestion	EASYnLC 1000	Q-Exactive	84 min	Mascot Distiller, v2.7.0	UniProt	M oxidation	<1%	Yes
17	Trypsin using the manufacturer's protocol	In-gel digestion	Thermo Dionex Ultimate 3000RSLCnano	Elite	60 min	Mascot	NCBI	M oxidation, up to 5 missed cleavages were allowed.	NR	No

*NR=not reported.

The subsequent sections provide a comprehensive description of the materials and methods, along with the specifics of sample preparation and LC-MS/MS analysis, as contributed by each participating proteomic center. These contributions are presented essentially as received, with minor modifications implemented to ensure consistency in units and similar parameters. This study proudly encompasses contributions from 17 distinct centers, each bringing unique expertise and insights to the research, including Case western Reserve University (Link), University of Cincinnati (Link), Cornell University (Link), Harvard University (Link), University of Kansas Medical School (Link), University of Missouri (Link), Massachusetts Institute of Technology (Link), Stanford University (Link), University of Tennessee (Link), University of California San Diego (Link), University of Nebraska–Lincoln (Link), Wayne State University (Link), University of Illinois (Link), University of Florida (Link), University of Nevada Reno (Link), Michigan State University (Link), and University of Texas at San Antonio (Link) blindly numbered from 1 to 17. The descriptions provided below regarding the preparation of LC-MS/MS samples have been reproduced from our recent publication[1].

Center #1: To initiate the digestion of bead-bound proteins, a 10 µL aliquot of trypsin (10 ng/µL) in 100 mM ammonium bicarbonate was added directly to the washed beads, maintaining an enzyme-to-protein ratio of 1:100 (wt/wt). To ensure thorough suspension of the beads in the protease solution, the samples were vortexed for 15 seconds at intervals of 2–3 minutes during the initial 15 minutes. The digestion process was then continued overnight at 37 °C in an oven incubator, without further stirring of the beads. Following this period, an additional 10 µL of protease was introduced to each sample, and the digestion was extended for 4 more hours at 37 °C. The tubes were subsequently placed on a magnetic rack to separate the supernatant, which was then removed.

For the final preparation step, the digested samples were diluted with 100% formic acid to achieve a concentration of 5% formic acid (vol/vol). These samples were purified using a C18 ultra micro spin column as per the manufacturer's instructions. Post-cleaning, the samples were dried using a SpeedVac and reconstituted in 1% acetic acid for analysis.

The LC-MS analysis was performed using a Dionex Ultimate 3000 nano-flow HPLC system interfaced with a ThermoScientific Fusion Lumos mass spectrometer. The HPLC setup included an Acclaim PepMap 100 precolumn (75 µm x 2 cm, C18, 3 µm, 100 A), followed by an Acclaim PepMap RSLC analytical column (75 µm x 15 cm, C18, 2 µm, 100 A). We injected 5 µL of the sample, and the peptides were eluted using an acetonitrile/0.1% formic acid gradient at a flow rate of 0.3 µL/min, subsequently introduced into the mass spectrometer's source online. Operating at 2.5 kV, the micro-electrospray ion source facilitated the analysis of the digest, which was performed using the instrument's data-dependent multitask capability. This process involved acquiring full scan mass spectra to determine peptide molecular weights and product ion spectra for amino acid sequencing in successive scans.

The resultant data were searched against the human SwissProtKB protein database using Sequest and MSFragger programs. Protein and peptide validations were carried out with Scaffold to <1% False Discovery Rate (FDR) at both the protein and peptide levels.

Center #2: A 125 µL aliquot of the provided sample in PBS was transferred into a separate tube and digested overnight at 37 °C using 50 ng of Promega porcine trypsin. The reaction was quenched by adding 5% formic acid to achieve a final concentration of 0.5%. The resultant peptides were then dried using a SpeedVac and reconstituted in 0.1% formic acid. Subsequent desalting and concentration were carried out using the C18 stage tip method.2 The eluate from the stage tip was dried, reconstituted in 20 µL of 0.1% formic acid, and injected in three technical replicates of 5.5 µL each for nanoLC-MS/MS analysis.

Mass spectrometry data collection was performed using an Orbitrap Eclipse mass spectrometer coupled to a Dionex Ultimate 3000 RSLCnano system (ThermoFisher Scientific). Samples were loaded onto a 5 mm nanoviper µ-Precolumn (i.d. 300 µm, C18 PepMap 100, 5.0 µm, 100 Å) from ThermoFisher Scientific at a flow rate of 5 µl/min in 0.1% formic acid in water for 5 minutes for desalting and concentration purposes. The trap-column was then switched in-line with an EASY-Spray column PepMap RSLC C18 (150 mm, i.d. 75 µm, C18, 3.0 µm, 100 Å). Peptides were eluted over a 60-minute gradient, varying from 98% phase A (0.1% formic acid in water) to 32% phase B (0.1% formic acid in acetonitrile), at a flow rate of 300 nL/min.

MS1 data were acquired in the Orbitrap at a resolution of 120,000, with a maximum injection time of 50 ms and an AGC target of 4 × 10^5. Only charge states from 2 to 6 were selected for MS2 analysis, using a 20-second dynamic exclusion window. The cycle time was set to 2.5 seconds. MS2 scans were conducted in the ion trap using HCD fragmentation (isolation window 0.8 Da; NCE 30%; maximum injection time 40 ms; AGC 5 × 10^4). The data were captured using Xcalibur 4.3 software (ThermoScientific).

Protein identification and peptide intensities were analyzed using Proteome Discoverer 2.4 (ThermoScientific), searched against the UniProt human database (UP000005640, downloaded on 12/11/2020) with the Sequest HT search algorithm. A modified LFQ standard processing and consensus workflow was employed. The workflow included mass recalibration (spectrum files RC), standard spectrum selector, minora feature detector, Sequest HT, and Percolator nodes. The precursor detector node was utilized to minimize chimeric spectra. Settings included a precursor mass tolerance of 10 ppm and fragment mass tolerance of 0.02 Da, allowing for up to 2 missed trypsin cleavages, and variable peptide modifications (oxidized methionine, N-terminal acetylation, and methionine loss). FDR tolerances were set to 0.01 for high confidence and 0.05 for medium confidence in the Percolator node. Peptide intensities were extracted from MS1 ion profiles of confidently identified proteins and tabulated as abundances across the three technical replicates.

Center #3: The in-solution digestion of the sample was carried out using an S-Trap micro spin column (ProtiFi, Huntington, NY, USA), following the S-Trap protocol as previously described[3-4], with some modifications. Initially, the sample solution underwent centrifugation at 12,000xg for 5 minutes to pellet the beads. The supernatant PBS storage solution was carefully pipetted off and retained for future use.

Subsequently, the bead pellets were incubated at room temperature for 30 minutes in a 25 µL buffer. This buffer was composed of 50 mM triethylammonium bicarbonate (TEAB) at pH 8.5, 6 M Urea, 2 M Thiourea, 4% SDS, and 10 mM Dithiothreitol (DTT). The sample was centrifuged once more at 12,000xg for 5 minutes to re-pellet the beads. The supernatant was then removed and incubated at 34 °C for an additional 30 minutes to ensure complete reduction. The reduction step was followed by alkylation using 65 mM iodoacetamide for 30 minutes in the dark and subsequently quenched with 35 mM DTT.

After the quenching step, the sample was acidified with 12% phosphoric acid to reach a final concentration of 1.2%. This was then diluted in a 1:7 ratio (v/v) with a solvent consisting of 90% methanol and 0.1 M TEAB at pH 8.5. The diluted sample was then transferred to an S-Trap micro unit and centrifuged at 3000xg for 30 seconds. The spin column was washed thrice using 150 µl of a solution of 90% methanol and 0.1 M TEAB at pH 8.5.

Digestion was carried out by adding 25 µl of trypsin (40 ng/µl) in 50 mM TEAB at pH 8.5 onto the spin column. The column was then incubated overnight (approximately 16 hours) at 37 °C. Post-incubation, the digested peptides were eluted from the S-Trap column in a sequential manner using 40 µl each of 50 mM TEAB at pH 8.5, followed by 0.2% formic acid, and finally 50% acetonitrile with 0.2% formic acid. The three eluates containing the eluted peptides were pooled and subsequently evaporated to dryness using a Speedvac SC110 (Thermo Savant, Milford, MA).

The tryptic digest was reconstituted in 73 µL of 0.5% formic acid (FA) and divided into three separate vials for nanoLC-ESI-MS/MS analysis. This analysis was performed using an Orbitrap Fusion Tribrid mass spectrometer (Thermo-Fisher Scientific, San Jose, CA)[5] equipped with a nanospray Flex Ion Source. The system was coupled with a Dionex UltiMate 3000 RSLCnano system (Thermo, Sunnyvale, CA).5 For each sample, 20 µL aliquots were injected onto a PepMap C-18 RP nano trapping column (5 µm, 100 µm i.d. x 20 mm) at a flow rate of 20 µL/min for rapid sample loading. The samples were then separated on a PepMap C-18 RP nano column (2 µm, 75 µm i.d. x 25 cm) maintained at 35 °C.

The tryptic peptides underwent elution in a 90-minute gradient ranging from 5% to 35% ACN in 0.1% formic acid at a flow rate of 300 nL/min. This was followed by a 7-minute increase to 90% ACN-0.1% FA and an 8-minute hold at this composition. Prior to each run, the column was re-equilibrated with 0.1% FA for 25 minutes. The Orbitrap Fusion operated in positive ion mode, with the spray voltage set at 1.1 kV and source

temperature at 275 °C. External calibration was performed for FT, IT, and quadrupole mass analyzers.

In the data-dependent acquisition (DDA) analysis, the instrument utilized the FT mass analyzer for MS scans to select precursor ions, followed by 3-second "Top Speed" data-dependent CID ion trap MS/MS scans with 1.6 m/z quadrupole isolation for precursor peptides. These peptides were identified based on multiple charged ions exceeding a threshold ion count of 10,000 and normalized collision energy of 30%. MS survey scans were conducted at a resolving power of 120,000 (fwhm at m/z 200), covering a mass range of m/z 375-1575. Dynamic exclusion parameters were set to a 50-second duration with ±10 ppm exclusion mass width. All data were acquired using Xcalibur 4.4 operation software (Thermo-Fisher Scientific).

The DDA raw files, containing MS and MS/MS data, were processed using Proteome Discoverer (PD) 2.4 software (Thermo Fisher Scientific, Bremen, Germany) with the Sequest HT algorithm. The PD 2.4 processing workflow, which included the Minora Feature Detector for precursor ion-based quantification, facilitated protein identification and relative quantitation of identified peptides and their modifications. The database search was conducted against a Homo Sapiens NCBI database, setting the peptide precursor tolerance to 10 ppm and fragment ion tolerance to 0.6 Da. Variable modifications such as methionine oxidation, deamidation of asparagine/glutamine, acetylation, methionine loss, and methionine loss plus acetylation on protein N-termini, along with fixed cysteine carbamidomethylation, were included in the search. Only high-confidence peptides, as defined by Sequest HT with a 1% FDR by Percolator, were considered for confident peptide identification. Relative quantitation of identified proteins across the three replicates was achieved using the Label-Free Quantitation (LFQ) workflow in PD 2.4, summing the unique plus razor peptides for each protein in each replicate to calculate protein abundance.

Center #4: A 10 µl aliquot of modified sequencing-grade trypsin (20 ng/µl, Promega, Madison, WI) was added to 300 µl of PBS, and the samples were incubated overnight at 37 ºC. To acidify the samples, 20 µl of a 20% formic acid solution was introduced, followed by desalting using the STAGE tip method.[2] On the day of analysis, the samples were reconstituted in 10 µl of HPLC solvent A.

For the chromatographic separation, a nano-scale reverse-phase HPLC capillary column was prepared by packing 2.6 µm C18 spherical silica beads into a fused silica capillary (100 µm inner diameter x ~30 cm length) with a flame-drawn tip.[6] The column was equilibrated before each sample was loaded using a Famos auto sampler (LC Packings, San Francisco, CA). A gradient was formed using increasing concentrations of solvent B (97.5% acetonitrile, 0.1% formic acid), which facilitated the elution of peptides.

As the peptides eluted, they were ionized via electrospray ionization before being introduced into an LTQ Orbitrap Velos Elite ion-trap mass spectrometer (Thermo Fisher Scientific, Waltham, MA). This process enabled the detection, isolation, and fragmentation of peptides, producing a tandem mass spectrum for each peptide's specific fragment ions. The peptide sequences, and consequently the protein identities, were determined by matching the acquired fragmentation patterns to protein databases using the Sequest software program (Thermo Fisher Scientific, Waltham, MA).[7] The databases used included a reversed version of all sequences, and the data were filtered to achieve a peptide false discovery rate of between one and two percent.

Center #5: For protein digestion, the coated particles were initially washed twice with 50 µl of 100 mM ammonium bicarbonate solution and then transferred to a new tube for two additional washes with the same solution. Following the final centrifugation, the protein-coated particles were incubated with 10 ng of sequencing-grade modified trypsin (Promega) in 50 µl. After a brief vortex, the mixture was incubated overnight at 37 °C. Subsequently, the coated particles were centrifuged using a tabletop centrifuge. An additional wash was performed with 50 µl of 100 mM ammonium bicarbonate, after which the supernatants were collected and acidified with formic acid to a final concentration of 5%.

For HPLC online tandem mass spectrometry analysis, the digested peptide mixture was divided into three aliquots and resuspended in 10 µL of 0.1% formic acid. Each aliquot was loaded onto a reversed-phase C18 peptide trap column (Aclaim PepMap 75 µm x 2 cm, 3 µm, 100Å) and washed for 10 minutes with 0.1% formic acid. The peptide trap was then connected to a reversed-phase C18 column (Aclaim PepMap 50 µm x 15 cm, 3 µm, 100Å) coupled online to an Orbitrap Fusion Lumos Tribrid mass spectrometer. The peptides were eluted using a gradient of solvent B (80% acetonitrile, 0.1% formic acid) in solvent A+B (solvent A: 0.1% formic acid, 3% acetonitrile), increasing from 2% to 25% B over 70 minutes, 25% to 40% B over 15 minutes, and 40% to 90% B over 1 minute, followed by a 10-minute wash with 90% B. The eluting peptides were ionized by ESI at 2700 volts, with the transfer tube temperature set to 275 °C. MS data acquisition was conducted in data-dependent mode on a 3-second cycle. MS scans were acquired in profile mode in the m/z range of 375 to 1500 in the Orbitrap analyzer at 120,000 resolution, using a 50 ms maximum scan time and 120 s exclusion time, with a mass tolerance of 10 ppm. Tandem mass scans were obtained via HCD at 35% collision energy in centroid mode, with Orbitrap detection at 30,000 resolution and a maximum ion time of 54 ms.

Data analysis was performed using Protein Discoverer v.2.4 running Sequest HT against a human protein database (downloaded from UniProt on 7/2/2019). Full trypsin specificity was set with a maximum of three missed cleavages, a precursor mass tolerance of 20 ppm, and a fragment tolerance of 0.02 Da. Variable modifications such as Met oxidation, deamidation of Asn and Gln, phosphorylation of S, T, and Y, and

Acetyl protein N-terminal modification were defined, allowing a maximum of three equal modifications and up to four dynamic modifications. For false discovery rate (FDR) assessment, a concatenated reversed protein database was utilized. Peptide and protein FDR were set at 5%, with validation based on the q-value obtained from Percolator and a p significance set at 0.05.

Center #6: All reagents used in this procedure were sourced from Fisher Scientific. To enhance the efficiency of digestion and maximize proteome yield, the nanoparticles (NPs) were initially concentrated by centrifugation at 16,000 x g for 20 minutes, after which the supernatant was carefully transferred to a fresh tube. The NP pellet was then resuspended in 20 µl of urea buffer (6 M urea, 2 M thiourea, 100 mM ammonium bicarbonate, pH 8.0). Simultaneously, the supernatant received an addition of 1/10th volume of the same urea buffer. Both samples underwent reduction, alkylation, and digestion with 1 µg of trypsin (Promega V5111; Lot# 0000475640), as per our Standard Operating Procedure (available upon request). Post-digestion, the peptides were desalted and concentrated using C18 100 µL tips (Pierce Cat# 87784; Lot#wa319454), lyophilized, and subsequently resuspended in 10 µL of 5% ACN, 0.1% FA. It is important to note that the resuspension volume used for the supernatant resulted in an approximate 30-fold concentration of the sample.

For the LC-MS/MS analysis, 1 µl injections were made in triplicate directly onto a 20 cm long x 75 µm inner diameter pulled-needle analytical column packed with Waters BEH-C18, 1.7 µm reversed phase resin. The peptides were separated and eluted from the analytical column using a gradient of acetonitrile at a flow rate of 300 nl/min. The LC system, a Bruker nanoElute, was interfaced with a Bruker timsTOF-PRO mass spectrometer via a Bruker CaptiveSpray source.

The LC gradient conditions were as follows: Starting at 3% B (A: 0.1% formic acid in water, B: 99.9% acetonitrile, 0.1% formic acid), the gradient increased to 17% B over 15 minutes, followed by a ramp to 25% B over 25 minutes, 37% B over 15 minutes, and finally 80% B over 10 minutes. The column was then held at 80% B for 20 minutes before returning to the initial conditions over 1 minute and holding for an additional 4 minutes. The total runtime for each analysis was 90 minutes. (Internal reference: “Notrap_90min_BEHC18_20cmx75um”).

Mass spectrometry data were collected in positive-ion data-dependent PASEF mode over an m/z range of 100 to 1700, with the last calibration dated 11/23/2021. Both PASEF and TIMS were activated. Each cycle, lasting 1.1 seconds, consisted of one MS and ten PASEF frames (~1 MS and 120 MS/MS). The target MS intensity was set at 10,000 counts/sec, with a minimum threshold of 1,000 counts/s. A charge-state-based rolling collision energy table ranged from 76-123% of 42.0 eV. An active exclusion/reconsider precursor method with a 0.4-minute release was employed. If a precursor was detected with >4X signal intensity in subsequent scans (within a mass width error of 0.015 m/z), a second MS/MS spectrum was collected. The isolation width

was set to 2 m/z for masses less than 700 m/z and 3 m/z for 800-1500 m/z. (Internal reference: “StandardPASEF_Cal-2021”).

Data analysis was conducted on our PEAKS-XPro server against the UniProt-Human database (20,379 entries; last updated 03/08/2021) using the following parameters: trypsin semi-specific as the enzyme, allowance of 2 missed cleavages; 20 ppm mass error on precursor, 0.1 Da mass error on CID MS/MS fragments; carbamidomethyl-Cys as a fixed modification; and oxidized-Met, deamidated-N/Q as variable modifications. The data were filtered to ensure a peptide false discovery rate of $p<0.1$; protein groups were shown exclusively, removing redundant database entries, and a minimum of 2 spectral counts was required.

Center #7: The samples were adjusted to a total volume of 100 µL with 6 M urea and 100 mM Tris (pH 7.8). Reduction and alkylation of disulfide bonds were then performed sequentially. Initially, 5 µl of 200 mM dithiothreitol (DTT) was added and incubated for 60 minutes to reduce the disulfide bonds. Subsequently, alkylation of the newly freed cysteine residues was carried out by adding 20 µl of 200 mM iodoacetamide and incubating for another 60 minutes, leading to the formation of carbamidomethyl cysteine. The urea concentration was then diluted by bringing the solution to a total volume of 900 µl.

To this diluted solution, 100 µl of trypsin or chymotrypsin (20 ng/µl) was added, achieving a final volume of 1000 µl. This mixture was digested overnight at 37 °C with gentle shaking. The resulting peptides were then washed, extracted, and concentrated using Waters Sep-Pak Plus C18 cartridges. Organic solvents were removed, and the volume was reduced to 50 µl using a speed vac for subsequent analyses. Three 10 µl aliquots of the digested extract were analyzed by reversed-phase high-performance liquid chromatography (HPLC) using Waters NanoAcquity pumps and autosampler, coupled with a ThermoFisher Orbitrap Elite mass spectrometer in a nano flow configuration.

For peptide trapping and washing, a 20 mm x 180 µm column packed with 5 µm Symmetry C18 material (Waters) was used at a flow rate of 15 µl/min for two minutes. The peptides were then eluted onto an analytical column, self-packed with 3.6 µm Aeris C18 material (Phenomenex) in a fritted 20 cm x 75 µm fused silica tubing with a 5 µm tip. The gradient consisted of 1% Buffer A (1% formic acid in water) isocratically for 1 minute at 250 nl/min, with increasing concentrations of Buffer B (1% formic acid in acetonitrile) to 15% B at 42.5 minutes, 27% B at 62 minutes, and 40% B at 73.5 minutes. The column was then washed and re-equilibrated between runs, with a total cycle time of approximately 97 minutes.

The mass spectrometer operated in data-dependent acquisition mode, selecting the 10 most abundant peptides detected in full scan mode in the Orbitrap at a resolution of 240,000 for fragmentation in the linear ion trap. An exclusion list of parent ions was maintained to enhance peptide diversity throughout the chromatographic run.

Peptides were identified using PEAKS Studio 10plus (Bioinformatic Solutions, Inc) algorithms against Refseq entries for Homo sapiens downloaded from NCBI, concatenated with a database of common contaminants. An additional database of 60 sequences was also included. A peptide threshold of 99.5% was set as the cutoff for the identification of peptides and proteins.

Center #8: Prior to reduction and alkylation, nanoparticles were resuspended in 200 µl of 50 mM triethylammonium bicarbonate (TEAB). Reduction of disulfide bonds was achieved with 10 mM dithiothreitol (DTT), followed by alkylation using 30 mM acrylamide to cap cysteine residues. Digestion was carried out using a combination of Trypsin/LysC (Promega) and 0.02% ProteaseMax (Promega) and was allowed to proceed overnight. Post-digestion, the peptides were quenched, desalted, dried, and reconstituted in 2% aqueous acetonitrile in preparation for mass spectrometry analysis.

Mass spectrometry experiments were conducted on a Q Exactive HF-X Hybrid Quadrupole - Orbitrap mass spectrometer (Thermo Scientific, San Jose, CA) coupled with a Nanoacquity UPLC system (Waters Corporation, Milford, MA). The UPLC system was calibrated to a flow rate of 300 nl/min, utilizing mobile phase A (0.2% formic acid in water) and mobile phase B (0.2% formic acid in acetonitrile). The analytical column, fabricated in-house, featured an internal diameter of 100 microns tapered to a nanospray emitter using a P2000 laser puller (Sutter Instrument, Novato, CA). This column was packed with NanoLCMS solutions 1.8 µm C18 stationary phase to an approximate length of 25 cm. The peptide injection was followed by an elution gradient ranging from 2-45% mobile phase B, plus a high-B wash, over a total of 80 minutes. The mass spectrometer was set to operate in data-dependent mode, utilizing HCD fragmentation for generating MS/MS spectra.

For data analysis, RAW files were processed using Byonic v4.2.4 (Protein Metrics, Cupertino, CA). A concatenated FASTA file, including UniProt Homo sapiens sequences along with sequences of other common contaminants and impurities, was used as the reference database. Semi-specific proteolysis by Trypsin/LysC, allowing for N-ragged cleavage with up to two missed cleavage sites, was assumed. Both precursor and fragment mass accuracies were maintained within 12 ppm. In the search parameters, cysteine modified with propionamide was set as a fixed modification, while variable modifications included oxidation on methionine, histidine, and tryptophan, dioxidation on methionine and tryptophan, deamidation on asparagine and glutamine, and acetylation on the protein N-terminus. Protein identifications were validated to a false discovery rate of 1% using the standard reverse-decoy technique.[8]

Center #9: A 250 µl aliquot of the sample suspension was combined with 25 µl of 10% SDS, resulting in a final SDS concentration of approximately 0.91%. This mixture was then incubated at 50 °C for 30 minutes to facilitate protein extraction. After

cooling to room temperature (RT), it was centrifuged at 16,000xg for 20 minutes at RT. Subsequently, 240 µl of the supernatant was carefully collected for further analysis. It is assumed that this 240 µl protein extract contains up to, but not exceeding, 6 µg of protein.

The collected protein extract was then mixed with 13 µl of 1 M triethylammonium bicarbonate (TEAB), achieving a final concentration of approximately 50 mM. The protein extraction underwent reduction with 5 mM DTT at 50 °C for 45 minutes, followed by alkylation with 20 mM iodoacetamide (IAM) at RT for 20 minutes in the dark. For protein precipitation, 1350 µl of precooled acetone was added to the mixture, which was then left overnight at -20°C. After centrifugation at 16,000xg for 10 minutes at 4°C, the resultant protein pellet was collected.

This pellet was redissolved in 200 µl of 10 mM TEAB, and protein precipitation was repeated overnight at -20°C with the addition of 1000 µl of precooled acetone. The air-dried protein pellet was then reconstituted in 75 µl of 100 mM TEAB. Proteins were digested overnight at 37 °C using 0.4 µg of Pierce Trypsin/Lys-C Protease mixture (cat. # A41007, Thermo Fisher). The digested material was vacuum-dried and desalted using Pierce C-18 spin tips (cat. # 84850), following the manufacturer's instructions. The desalted peptide sample was further dried in a SpeedVac for 60 minutes and reconstituted in 50 µl of loading buffer (3% acetonitrile with 0.1% TFA) for LC/MS/MS analysis.

For peptide and protein identification and label-free quantitation, 5 µl aliquots (containing less than 0.6 µg) of the digested and desalted material were analyzed in triplicate using HPLC inline with tandem mass spectrometry. The specific instrumentation and parameters used for this analysis are detailed below:

HPLC: Ultimate 3000RSLCnano, Thermo Fisher

Trap column: Acclaim PepMap 100, 75µm x 20mm, C18, 3µm, 100Å, Thermo Fisher

Column: Acclaim PepMap RSLC, 75µm x 500mm (ID x Length), C-18, 2µm, 100Å, Thermo Fisher

Injection volume/mode: 5µl/µl PickUp

Loading Buffer: 3% acetonitrile with 0.1% TFA

Loading flow rate and duration: 5µl/min for 5min

Solvent A: 0.1% formic acid in water, LC/MS grade, Thermo Fisher

Solvent B: 0.1% formic acid in acetonitrile, LC/MS grade, Thermo Fisher

LC flow rate: 300 nl/min

Column temperature: 40 °C

Gradient: 0min-3%B, 4min-3%B, 5min-5%B, 55min-25%B, 60min-30%B, 63min-90%B, 73min-90%B, 76min-3%B, 100min-3%B

MS: Orbitrap Fusion Lumos, Thermo Fisher

Data dependent analysis (DDA): 3sec cycles

MS scan (full): Analyzer - Orbitrap, resolution-120,000 (FWHM, at m/z=200)

Scan Filters: MIPS mode - Peptide

Intensity threshold ≥ 10,000

Charge state - 2-6

Dynamic exclusion – 30 sec

MS2 scan (full): Quadrupole isolation window - 0.7 m/z,

Activation - HCD (30%)

Analyzer - Orbitrap, Resolution 30,000 (FWHM, at m/z=200)

Post-Acquisition Analysis

Proteome Discoverer 2.4, Thermo Fisher

`Peptide/Protein Identification

Search engine: Sequest HT

Database: SwissProt, TaxID 9606 (Homo sapiens), v.2017-05-10, 42153 entries

Enzyme: Trypsin (full)

Dynamic modification: Oxidation of Met, acetylation of the protein N-terminus

Static modification: Carbamidomethylation of Cys

Precursor and fragment ion mass tolerance: 10 ppm and 0.02 Da, respectively

Validation and filtering at PSM level (q value): Percolator, FDR ≤0.01

Identification of protein or protein group: At least one validated peptide sequence unique to a protein or a protein group

Protein groups: Strict parsimony principle applied

Validation at protein level (Experimental q value): strict - FDR≤0.01, relaxed - FDR≤0.05

Feature Detection

Min Trace Length: 5

Min # Isotopes: 2

Max ΔRT of Isotope Pattern Multiplets: 0.2min

Chromatographic Alignment

Max RT shift: 5 min

Mass tolerance: 10 ppm

Feature Linking/Mapping

RT tolerance: 0 (automatic)

Mass tolerance: 0 (automatic)

Min S/N threshold: 5

Peptide/Protein Quantification:

Quantification: LFQ - Label-free Quantification (Precursor Ion Area Detection)

Peptides to use: Unique + Razor

Peptide uniqueness: Protein Group

Peptide Abundance: MS Peak Area

Normalization mode: Total Peptide Amount

Peptide abundance: Summed abundances of assigned PSMs

Protein abundance: Summed abundances of assigned peptides

Peptide Group abundance: Mean of replicate abundances

Protein Group abundance: Mean of replicate abundances

Center #10: Protein samples were appropriately diluted in TNE buffer (50 mM Tris pH 8.0, 100 mM NaCl, 1 mM EDTA). To facilitate denaturation, RapiGest SF reagent (Waters) was added to achieve a final concentration of 0.1%, and the samples were then boiled for 5 minutes. Subsequently, TCEP (Tris (2-carboxyethyl) phosphine) was introduced to a final concentration of 1 mM, and the samples were incubated at 37 °C for 30 minutes. The reduction step was followed by alkylation using 0.5 mg/ml of iodoacetamide for 30 minutes at 37 °C, and then neutralization with 2 mM TCEP (final

concentration).[9]

The protein samples were then digested overnight at 37 °C using trypsin at a trypsin-to-protein ratio of 1:50. Post-digestion, RapiGest was degraded and removed by treating the samples with 250 mM HCl at 37 °C for 1 hour, followed by centrifugation at 14,000 rpm for 30 minutes at 4 °C. The supernatant was transferred to a new tube, and peptides were extracted and desalted using C18 columns (Thermo Scientific, PI-87782). Peptide quantification was performed using the BCA assay, and 1 µg of peptides was subsequently injected for LC-MS analysis.9

The trypsin-digested peptides were analyzed using ultra-high pressure liquid chromatography (UPLC) coupled with tandem mass spectroscopy (LC-MS/MS) and nano-spray ionization. The Orbitrap Fusion Lumos hybrid mass spectrometer (Thermo) interfaced with nano-scale reversed-phase UPLC (Thermo Dionex UltiMate™ 3000 RSLC nano System) was employed for this analysis. The column used was a 25 cm, 75-micron ID glass capillary packed with 1.7-µm C18 (130) BEHTM beads (Waters corporation). Peptides were eluted into the mass spectrometer using a linear gradient of 5–80% ACN over 1.5 hours at a flow rate of 375 µl/min. The gradient was formed using Buffer A (98% H2O, 2% ACN, 0.1% formic acid) and Buffer B (100% ACN, 0.1% formic acid).

The mass spectrometer parameters were as follows: an MS1 survey scan was conducted using the orbitrap detector (mass range (m/z): 400-1500, 120,000 resolution, quadrupole isolation) with a spray voltage of 2200 V, ion transfer tube temperature of 275 °C, AGC target of 400,000, and a maximum injection time of 50 ms. Data-dependent scans targeted the most intense ions (+2 to +5 charge states) with a 5-second exclusion time, selecting ions with a minimum intensity of 50,000. Collision events were executed in the high-energy collision cell (HCD, collision energy of 30%), and the fragment masses were analyzed in the ion trap mass analyzer (ion trap scan rate of turbo, first mass m/z 100, AGC target 5000, maximum injection time of 35ms). Protein identification was conducted using Peaks Studio 8.5 (Bioinformatics Solutions Inc).[10]

Center #11: The sample was initially centrifuged at 21,000 x g for 10 minutes, and the PBS supernatant was carefully removed. Subsequently, 50 µl of 1X NuPAGE™ LDS sample buffer (containing 5 mM DTT as a reducing agent) was added to resuspend the bead pellet. The suspension was then heated at 95 °C for 10 minutes with gentle shaking to ensure thorough denaturation. After a repeat centrifugation at 21,000 x g for 10 minutes, the supernatant was separated from the beads. Of the 50 µl, 45 µl was loaded onto a 12% BOLT™ SDS-PAGE gel (Bolt™ 12%, Bis-Tris, 1.0 mm, Mini Protein Gel, 10-well, ThermoFisher Scientific) and run to the top 1 cm of the gel.

The gel section containing the protein was excised, reduced with DTT, and alkylated with iodoacetamide. Following this, the gel samples underwent washing, destaining, and overnight digestion at 37 °C with 250 ng of trypsin (Promega sequencing grade, cat# V5111). The peptides were extracted from the gel pieces, dried down, and

re-dissolved in 30 µl of a solution containing 5% acetonitrile and 0.5% trifluoroacetic acid. For nanoLC-MS/MS analysis, three 5 µl aliquots were run using a 2-hour method on a CSH 0.075 mm x 250 mm C18 column (Waters Corp, Milford, MA), which fed into an Orbitrap Eclipse mass spectrometer (Thermo Fisher) operating in OT-OT mode.

Protein quantitation was performed using Proteome Discoverer (Thermo Fisher, version 2.4). All MS/MS samples were searched using Mascot (Matrix Science, London, UK; version 2.7.0), set up to search the cRAP_20150130.fasta (125 entries) and UniProt-human_20210508 databases (77,027 entries), assuming trypsin digestion. Mascot searches were conducted with a fragment ion mass tolerance of 0.02 Da and a parent ion tolerance of 10.0 PPM. Variable modifications included deamidation of asparagine and glutamine, and oxidation of methionine, while carbamidomethylation of cysteine was a fixed modification. Peptides were validated by Percolator with a posterior error probability (PEP) threshold of 0.01. A decoy database search was employed to set the false discovery rate at 1% (high confidence).

Quantification was based on precursor abundance, using intensity for peak abundance determination, and normalized against the total peptide amount. The peak abundances of each sample were adjusted relative to the maximum sum of all files, with the normalization factor being the ratio of the sum of each sample to this maximum. Scaled abundances were provided, ensuring that the average abundance of each sample equated to 100. This approach allowed for a comprehensive and accurate quantification of the proteins in the samples.

Center #12: A 300 µl sample was divided into three equal aliquots of 100 µl each. To each aliquot, triethylammonium bicarbonate (TEAB) buffer (Honeywell Fluka, cat# 60-044-974) was added to achieve a final concentration of 20 mM. The reduction of the samples was facilitated by the addition of 5 mM DL-Dithiothretol (DTT, Sigma, cat# D5545), followed by a 30-minute incubation at 37 °C. Alkylation was then carried out by incorporating 15 mM Iodoacetamide (IAA, Sigma, cat# I1149) into each sample, which was then incubated at room temperature for 30 minutes in the dark. To quench the alkylation reaction, an additional 5 mM DTT was added to each sample.

The trypsin digestion process was initiated by adding 0.35 µg of trypsin (Promega, V5113) to each aliquot, followed by incubation for 1 hour at 47 °C and subsequently for 3 hours at 37 °C. To assess the efficiency of digestion, a small portion of each sample was analyzed using LC-MS/MS. The final analysis was performed on the same samples utilizing a Thermo Scientific Easy-nLC 1000 chromatography system. The setup included a C18 Acclaim PepMap 100 trap column (75 µm x 2 cm) and an Acclaim PepMap RSLC column (75 µm x 25 cm), both from Thermo Scientific.

LC-MS/MS analysis was conducted using Data-Dependent Analysis on an Orbitrap Fusion MS system. MS1 spectra were acquired with a resolution of 120,000, while MS2 spectra were analyzed using the ion trap. The data were processed using Proteome Discoverer 2.4, employing Sequest NT and Percolator algorithms for database

searches. The human protein database used for the search was UniProt UP000005640, downloaded on 3/30/2021, containing 20,310 protein entries.

Search parameters allowed for up to 2 missed cleavages in trypsin digestion. Carbamidomethylation of cysteine was set as a fixed modification, while variable modifications included deamidation of asparagine and glutamine, oxidation of methionine, and N-terminal acetylation. The False Discovery Rate (FDR) was set at 0.01 for high-confidence peptide matches. This stringent FDR threshold ensured the reliability of the protein identifications obtained from the analysis.

Center #13:

The sample, prepared in 1X PBS, was digested with 0.5 micrograms of trypsin (Thermo Pierce, Proteomics Grade) for 30 minutes at 55 °C (60 watts) using a CEM Discover Microwave Digestor (Matthews, NC). Post-digestion, the material was lyophilized and then desalted using StageTips.[11] After a second lyophilization, the sample was reconstituted in a solution containing 5% acetonitrile and 0.1% formic acid in preparation for LC/MS analysis.

For the LC/MS analysis, 0.5 micrograms of the digested sample was utilized. The chromatographic separation was performed on a Thermo UltiMate 3000 UHPLC system, which was coupled online with a high-resolution Thermo Orbitrap Fusion Tribrid mass spectrometer. Peptide separation was achieved using reversed-phase chromatography on a 50 cm μPAC C18 nano-LC column (PharmaFluidics, Ghent, Belgium). The mobile phases consisted of 0.1% formic acid (A) and 0.1% formic acid in acetonitrile (B). A linear gradient, ranging from 4% B to 35% B over 90 minutes, was employed for effective peptide separation, followed by additional steps for column washing and regeneration. The mass spectrometer operated in a data-dependent mode, conducting precursor scans from 300 to 2000 m/z at a resolution of 120,000. This was followed by collision-induced dissociation of the most abundant precursors within a maximum cycle time of 3 seconds (35% NCE, 1.6 m/z isolation window, 60-second dynamic exclusion window).[11]

The resultant LC-MS/MS raw data was analyzed against the Swissprot database (version 2021-02) for Homo sapiens using Mascot 2.8 (London, UK). The analysis settings specified trypsin digestion with a maximum of two missed cleavages. Peptide and fragment mass tolerances were set at 10 ppm and 0.6 Da, respectively. Variable modifications in the search included the oxidation of methionine. This comprehensive analytical approach ensured accurate identification and quantification of the peptides and proteins in the sample.

Center #14: Total protein concentration in each sample was quantified using a Qubit fluorometer, and a volume corresponding to 8 μg of total protein was allocated for digestion. The samples were digested using the sequencing-grade trypsin/Lys-C rapid digestion kit from Promega (Madison, WI), following the manufacturer's recommended protocol. To each sample, three times its volume of rapid digestion buffer (supplied with the kit) was added. Samples were then incubated at 56 °C for 30 minutes with 1 μL of dithiothreitol (DTT) solution (0.1 M in 100 mM ammonium bicarbonate). This step was followed by the addition of 0.54 μL of 55 mM iodoacetamide

in 100 mM ammonium bicarbonate, incubated at room temperature in the dark for 30 minutes. The trypsin/Lys-C mix was freshly prepared at 1 µg/µL in the rapid digestion buffer, with 1 µL of enzyme added to each sample. The samples were then incubated at 70 °C for 1 hour. Digestion was quenched by adding 0.5% trifluoroacetic acid (TFA), and mass spectrometry analysis was conducted immediately to ensure the production of high-quality tryptic peptides with minimal non-specific cleavage.

Nano-liquid chromatography tandem mass spectrometry (Nano-LC/MS/MS) analysis was performed using a Thermo Scientific Q Exactive HF Orbitrap mass spectrometer equipped with an EASY Spray nanospray source, operated in positive ion mode. The chromatography system was an UltiMate™ 3000 RSLCnano from Thermo Scientific. Mobile phase A consisted of water with 0.1% formic acid, and mobile phase B was acetonitrile with 0.1% formic acid. For loading, mobile phase A contained 0.1% trifluoroacetic acid. Samples (5 µl) were injected onto a PharmaFluidics mPAC™ C18 trapping column (C18, 5 µm pillar diameter, 10 mm length, 2.5 µm inter-pillar distance) at a flow rate of 10 µl/min. The peptides were desalted and concentrated by washing with 1% B for 3 minutes. After switching the injector port, peptides were eluted from the trap onto a 50 cm PharmaFluidics mPAC™ column (C18, 5 µm pillar diameter, 50 cm length, 2.5 µm inter-pillar distance) maintained at 40 °C. An initial flow rate of 750 nl/min was used for the first 15 minutes, then reduced to 300 nl/min for the remainder of the run. The peptides were eluted into the Q Exactive HF Orbitrap system using a gradient of 1% B to 20% B over 100 minutes, followed by an increase to 45% B over the next 20 minutes. The total run time was 150 minutes.

**Time (min)**	**% B**	**Flow Rate (nL/min)**
0	1	750
3	1	750
15	5	750
15.1	5	300
100	20	300
123	45	300
130	95	300
135	95	300
135.1	1	300
150	1	300

The total runtime for the LC-MS/MS analysis was set at 150 minutes. Standard conditions established in our lab were followed for MS/MS acquisition. The EASY Spray source was operated with a spray voltage of 1.5 kV and a capillary temperature of 200 °C. The mass spectrometer's scan sequence was based on the established TopTen™ method, involving a full scan range of 375 – 1575 Da at 60,000 resolution. MS/MS scans

were performed at a resolution of 15,000, generating product ion spectra to determine amino acid sequences in consecutive instrument scans of the fifteen most abundant peaks in each spectrum.

The Automatic Gain Control (AGC) target ion number was set at 3e6 ions for the full scan and 2e5 ions for MS2 mode. Maximum ion injection times were set at 50 ms for full scans and 55 ms for MS2 mode, with the micro scan number fixed at 1 for both full and MS2 scans. The Higher-energy Collisional Dissociation (HCD) fragmentation energy was set to 28 with a stepped Normalized Collision Energy (NCE) and an isolation window of 4 m/z. Singly charged ions were excluded from MS2 scans. Dynamic exclusion was enabled with a repeat count of 1 within 15 seconds, and isotopes were excluded. A Siloxane background peak at 445.12003 Da served as the internal lock mass.

The integrity and performance of the LC columns and mass spectrometer were evaluated using a HeLa protein digest standard. Instrument maintenance, including cleaning and column replacement, was scheduled if the number of protein IDs from the HeLa standard fell below 2700.

For data analysis, all MS/MS samples were processed using Sequest (Thermo Fisher Scientific, San Jose, CA, USA; version IseNode in Proteome Discoverer 2.4.0.305). Sequest was configured to search the Homo sapiens database (NcbiAV TaxID=9606) (v2017-10-30), assuming trypsin digestion. The search parameters included a fragment ion mass tolerance of 0.020 Da and a parent ion tolerance of 10.0 ppm. Carbamidomethylation of cysteine was set as a fixed modification, while variable modifications included the loss and acetylation of methionine, oxidation of methionine, and acetylation of the N-terminus.

For protein identification, Scaffold (version Scaffold_4.11.1, Proteome Software Inc., Portland, OR) was utilized. Peptide identifications were accepted at greater than 95.0% probability as determined by the Percolator posterior error probability calculation. [12] Protein identifications were accepted at greater than 99.0% probability and required at least one identified peptide. Protein probabilities were assigned by the Protein Prophet algorithm. [13] Proteins with similar peptides, indistinguishable based on MS/MS analysis, were grouped according to the principles of parsimony, forming clusters based on shared peptide evidence.

Protein quantitation involved Fischer’s Exact test with a Benjamini-Hochberg correction for P-value calculation and fold change determination using weighted spectra.

Center #15: The protein sample underwent reduction and alkylation using iodoacetamide before being digested with a trypsin/Lys-C protease mixture. This preparation process was conducted using the Thermo Scientific EasyPep Mini MS

Sample Prep Kit (Cat #A40006), strictly adhering to the protocol provided with the kit. This ensured optimal preparation of the sample for subsequent mass spectrometry analysis.

Trap Method

No	Time	Flow [µl/min]	%B	%C	Curve
1	0.000	Equilibration			
2	0.000	10.000	0.0	100.0	5
3	*New Row*				
4	0.000	Run			
5	0.000	10.000	0.0	100.0	5
6	5.000	10.000	0.0	100.0	5
7	138.000	2.000	0.0	100.0	5
8	*New Row*				
9	138.000	Stop Run			

Separation Gradient

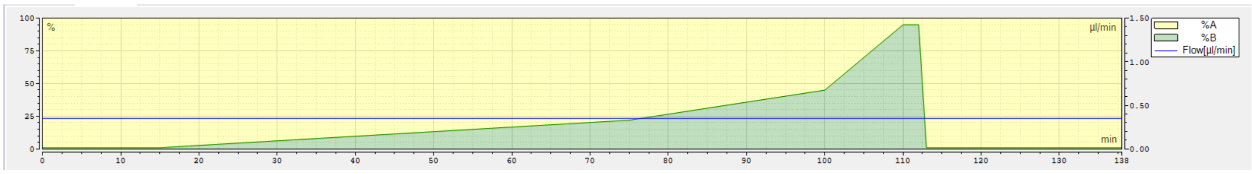

No	Time	Flow [µl/min]	%B	Curve
1	0.000	Equilibration		
2	0.000	0.350	1.0	5
3	*New Row*			
4	0.000	Run		
5	0.000	0.350	1.0	5
6	15.000	0.350	1.0	5
7	75.000	0.350	22.0	5
8	100.000	0.350	45.0	5
9	110.000	0.350	95.0	5
10	112.000	0.350	95.0	5
11	113.000	0.350	1.0	5
12	138.000	0.350	1.0	5
13	*New Row*			
14	138.000	Stop Run		

The sample preparation for analysis included a cleanup step using the column provided with the Thermo Scientific EasyPep Mini MS Sample Prep Kit. For the LC/MS analysis, the samples were processed using an UltiMate 3000 RSLCnano system (Thermo Scientific, San Jose, CA). Peptides were initially trapped on a 300 µm i.d. x 5 mm C18 PepMap 100 trap (Thermo Scientific, San Jose, CA) for 5 minutes at a flow rate of 10 µl/min. Subsequently, separation was carried out on a 50 cm µPAC C18 nano-LC column (PharmaFluidics, Ghent, Belgium) equipped with a 20 µm fused silica emitter and a Nanospray Easy-Spray ion source (Thermo Scientific, San Jose, CA). The separation

was conducted at a flow rate of 350 nl/min using a gradient from 1% to 45% solvent B (Solvent A: 0.1% Formic Acid, Solvent B: Acetonitrile, 0.1% Formic Acid).

Mass spectral analysis was performed on an Orbitrap Eclipse mass spectrometer (Thermo Scientific, San Jose, CA). The MS1 precursor selection range was set from 375-1500 m/z at a resolution of 120K with a normalized AGC target of 250% and automatic maximum injection time settings. Quadrupole isolation of 0.7 Th was used for MS2 isolation and CID fragmentation in the linear ion trap with a collision energy of 35% and a 10 ms activation time. The MS2 AGC was in standard mode with a 35 ms maximum injection time. The instrument operated in data-dependent mode with a 3-second cycle time, prioritizing the most intense precursors and employing dynamic exclusion with a duration of 60 seconds and a tolerance of 10 ppm.

For data analysis, all MS/MS samples were processed using Sequest (Thermo Fisher Scientific, San Jose, CA, USA; version IseNode in Proteome Discoverer 2.2.0.388). Sequest was configured to search the UniProt-homo_sapiens_20190201.fasta database (147857 entries), assuming trypsin digestion. The search parameters included a fragment ion mass tolerance of 0.60 Da and a parent ion tolerance of 10.0 PPM. Carbamidomethylation of cysteine was a fixed modification, while oxidation of methionine, N-terminal acetylation, and serine phosphorylation were variable modifications.

Validation of peptide and protein identifications was conducted using Scaffold (version Scaffold_5.0.1, Proteome Software Inc., Portland, OR). Peptide identifications were accepted at a probability greater than 95.0% as determined by the Peptide Prophet algorithm with Scaffold delta-mass correction.[14] Protein identifications required a probability greater than 95.0% and at least 2 identified peptides. Protein probabilities were assigned by the Protein Prophet algorithm.[15] Proteins sharing significant peptide evidence were grouped to adhere to the principles of parsimony.

Center #16: Protein-bound NPs in PBS were prepared for analysis with the addition of acetonitrile to a final concentration of 10%. Reduction of the sample was achieved by adding 10 mM Pierce BondBreaker TCEP. The mixture was then heated to 95 °C for 10 minutes with agitation at 700 rpm using an Eppendorf ThermoMixer R. After cooling to room temperature, the samples were alkylated by adding chloroacetamide to a final concentration of 15 mM and incubated at room temperature in the dark for 60 minutes. This alkylation step was quenched by the addition of DTT to 5 mM.

Digestion was performed by adding trypsin (dissolved in 50 mM ammonium bicarbonate) to a final concentration of 30 ng per sample, followed by overnight incubation at 37 °C. The solution was then acidified to 1% using trifluoroacetic acid and desalted using C18 solid phase extraction with StageTips.16 The purified peptide eluates were dried by vacuum centrifugation and stored at -20 °C.

For LC-MS analysis, the samples were reconstituted in a solution of 2% acetonitrile, 0.1% trifluoroacetic acid, and 97.9% water to a final volume of 12 µl. An automatic 10 µl injection was performed using a Thermo EASYnLC 1000 onto a Thermo Acclaim PepMap RSLC 0.1 mm x 20 mm C18 trapping column. The bound peptides were then eluted onto a Thermo Acclaim PepMap RSLC 0.075 mm x 250 mm resolving column over 95 minutes with a gradient starting from 5% B to 8% B at 2 minutes, 8% B to 28% B at 72 minutes, 28% B to 38% B at 84 minutes, increasing to 90% B at 85 minutes and held at 90% B for the remainder of the run. The mobile phases were Buffer A (99.9% water, 0.1% formic acid) and Buffer B (80% acetonitrile, 0.1% formic acid, 19.9% water), with a constant flow rate of 300 nl/min. The column temperature was maintained at 50 °C using an integrated column oven (PRSO-V1, Sonation GmbH, Biberach, Germany). Eluted peptides were analyzed using a Thermo Scientific Q-Exactive mass spectrometer with a FlexSpray ion source. Survey scans were conducted in the Orbitrap at 35,000 resolution (m/z 200), and the top 15 ions from each scan were subjected to HCD with fragment spectra acquired at 17,500 resolution.

MS/MS spectra were converted to peak lists using Mascot Distiller, v2.7.0, and searched against all human protein entries available from UniProt appended with common laboratory contaminants (cRAP project, thegpm.org). Scaffold, v5.1, was used for probabilistic validation of protein identifications, with assignments validated using the Scaffold 1% FDR confidence filter considered accurate. Mascot parameters included up to 2 missed tryptic sites, fixed modification of carbamidomethylation on cysteine, variable modification of oxidation of methionine, peptide tolerance of +/- 10 ppm, MS/MS tolerance of 0.02 Da, and FDR calculated using a randomized database search.

Center #17: A 100 µL aliquot of the sample was prepared by mixing it with an equal volume of 2x Laemmli sample buffer (Bio-Rad) containing 2-mercaptoethanol. This mixture was boiled for 5 minutes to ensure protein denaturation before being loaded onto a 12% Mini-PROTEAN® TGX™ Precast Gel. Electrophoresis was performed until the proteins migrated approximately 1 cm into the gel, followed by staining with Coomassie Brilliant Blue R-250. After destaining, the 1 cm bands were excised, pooled, and subjected to digestion using proteomics-grade trypsin (Sigma-Aldrich), adhering to the manufacturer's recommended protocol. The extracted tryptic peptides were then evaporated to dryness using a Speedvac and reconstituted in 15 µL of 0.1% aqueous formic acid in preparation for LC-MS/MS analysis.

For the analysis, three replicates of the trypsin-digested proteins were injected in 2 µL aliquots onto a nano-reversed-phase liquid chromatography (LC) electrospray ionization tandem mass spectrometry (MS) system. This system comprised an UltiMate 3000 Nano LC System and an LTQ-Orbitrap Elite mass spectrometer (Thermo Fisher, San Jose, CA). Chromatographic separation was performed using a home-made 25 cm × 75 µm ID column packed with XBridge™ BEH C18 beads (2.5 µm, 130 Å). The mobile phases, Solvent A (0.1% aqueous formic acid) and Solvent B (0.1% formic acid in acetonitrile), were employed to create an 85-minute gradient, which

included 60 minutes of 5-45% B, 15 minutes increasing from 45-90% B, and a final 10-minute hold at 90% B. The flow rate was maintained at 200 nL/min.

The LTQ-Orbitrap Elite mass spectrometer operated in positive ionization mode, with a 2.6 kV spray voltage and an ion transfer capillary temperature of 300 °C. Each MS and MS/MS scan was set to one microscan. A full scan MS range of 300 ≤ m/z ≤ 2000 was followed by data-dependent MS/MS scans of the 10 most intense ions. Full MS resolution was set at 60,000. Dynamic exclusion parameters were set with a repeat count of 1, a repeat duration of 30 seconds, and an exclusion duration of 30 seconds. Higher-energy collisional dissociation (HCD) was conducted at a normalized collision energy of 35%, with an activation time of 0.1 ms.

LC-MS/MS data files were converted to the mascot generic file format using ProteoWizard msConvert. The data was then searched against the NCBI human protein database using MASCOT (Matrix Science, London, UK) with search criteria that included a precursor ion mass tolerance of 25 ppm, a product ion mass tolerance of 50 mmu, allowance for up to 5 trypsin missed cleavages, and oxidation of methionine residues as a variable modification.[17]

Supplementary References

1. Ashkarran, A. A.; Gharibi, H.; Voke, E.; Landry, M. P.; Saei, A. A.; Mahmoudi, M., Measurements of heterogeneity in proteomics analysis of the nanoparticle protein corona across core facilities. *Nature Communications* **2022,** *13* (1), 6610.
2. Rappsilber, J.; Ishihama, Y.; Mann, M., Stop And Go Extraction tips for matrix-assisted laser desorption/ionization, nanoelectrospray, and LC/MS sample pretreatment in proteomics. *Analytical Chemistry* **2003,** *75* (3), 663-670.
3. Yang, Y.; Anderson, E.; Zhang, S., Evaluation of six sample preparation procedures for qualitative and quantitative proteomics analysis of milk fat globule membrane. *Electrophoresis* **2018,** *39* (18), 2332-2339.
4. Zougman, A.; Selby, P. J.; Banks, R. E., Suspension trapping (STrap) sample preparation method for bottom-up proteomics analysis. *Proteomics* **2014,** *14* (9), 1006-1000.
5. Harman, R. M.; He, M. K.; Zhang, S.; Van De Walle, G. R., Plasminogen activator inhibitor-1 and tenascin-C secreted by equine mesenchymal stromal cells stimulate dermal fibroblast migration in vitro and contribute to wound healing in vivo. *Cytotherapy* **2018,** *20* (8), 1061-1076.
6. Peng, J.; Gygi, S. P., Proteomics: The move to mixtures. *Journal of Mass Spectrometry* **2001,** *36* (10), 1083-1091.
7. Eng, J. K.; McCormack, A. L.; Yates, J. R., An approach to correlate tandem mass spectral data of peptides with amino acid sequences in a protein database. *Journal of the American Society for Mass Spectrometry* **1994,** *5* (11), 976-989.
8. Elias, J. E.; Gygi, S. P., Target-decoy search strategy for increased confidence in large-scale protein identifications by mass spectrometry. *Nature Methods* **2007,** *4* (3), 207-214.
9. Guttman, M.; Betts Gn Fau - Barnes, H.; Barnes H Fau - Ghassemian, M.; Ghassemian M Fau - van der Geer, P.; van der Geer P Fau - Komives, E. A.; Komives, E. A., Interactions of the NPXY microdomains of the low density lipoprotein receptor-related protein 1. (1615-9861 (Electronic)).
10. McCormack, A. L.; Schieltz Dm Fau - Goode, B.; Goode B Fau - Yang, S.; Yang S Fau - Barnes, G.; Barnes G Fau - Drubin, D.; Drubin D Fau - Yates, J. R., 3rd; Yates, J. R., 3rd, Direct analysis and identification of proteins in mixtures by LC/MS/MS and database searching at the low-femtomole level. (0003-2700 (Print)).
11. Rappsilber, J.; Mann M Fau - Ishihama, Y.; Ishihama, Y., Protocol for micro-purification, enrichment, pre-fractionation and storage of peptides for proteomics using StageTips. (1750-2799 (Electronic)).
12. Käll, L.; Storey, J. D.; Noble, W. S., Non-parametric estimation of posterior error probabilities associated with peptides identified by tandem mass spectrometry. *Bioinformatics* **2008,** *24* (16), i42-i48.
13. Hsu, J. L.; Huang Sy Fau - Chow, N.-H.; Chow Nh Fau - Chen, S.-H.; Chen, S. H., Stable-isotope dimethyl labeling for quantitative proteomics. (0003-2700 (Print)).
14. Keller, A.; Nesvizhskii Ai Fau - Kolker, E.; Kolker E Fau - Aebersold, R.; Aebersold, R., Empirical statistical model to estimate the accuracy of peptide identifications made by MS/MS and database search. (0003-2700 (Print)).
15. Nesvizhskii, A. I.; Keller, A.; Kolker, E.; Aebersold, R., A statistical model for identifying proteins by tandem mass spectrometry. *Analytical Chemistry* **2003,** *75* (17), 4646-4658.
16. Rappsilber, J.; Mann, M.; Ishihama, Y., Protocol for micro-purification, enrichment, pre-fractionation and storage of peptides for proteomics using StageTips. *Nature Protocols* **2007,** *2* (8), 1896-1906.
17. Chambers, M. C.; MacLean, B.; Burke, R.; Amodei, D.; Ruderman, D. L.; Neumann, S.; Gatto, L.; Fischer, B.; Pratt, B.; Egertson, J.; Hoff, K.; Kessner, D.; Tasman, N.; Shulman, N.; Frewen, B.; Baker, T. A.; Brusniak, M. Y.; Paulse, C.; Creasy, D.; Flashner, L.; Kani, K.; Moulding, C.; Seymour, S. L.; Nuwaysir, L. M.;

Lefebvre, B.; Kuhlmann, F.; Roark, J.; Rainer, P.; Detlev, S.; Hemenway, T.; Huhmer, A.; Langridge, J.; Connolly, B.; Chadick, T.; Holly, K.; Eckels, J.; Deutsch, E. W.; Moritz, R. L.; Katz, J. E.; Agus, D. B.; MacCoss, M.; Tabb, D. L.; Mallick, P., A cross-platform toolkit for mass spectrometry and proteomics. *Nature Biotechnology* **2012,** *30* (10), 918-920.
