# Supplementary material for: A uniform data processing pipeline enables harmonized nanoparticle protein corona analysis across proteomics core facilities
Source: Nat Commun. 2024 Jan 6;15:342. doi: 10.1038/s41467-023-44678-x (PMC10771434; doi:10.1038/s41467-023-44678-x)
Supplement: Supplementary file 3 — Description of Additional Supplementary Files [file 41467_2023_44678_MOESM3_ESM.pdf]

Title: Supplementary Data 1.
Description: Proteins quantified in identical nanoparticle protein corona samples across 15 core facilities.

Title: Supplementary Data 2.
Description: The 51 shared proteins across all 15 centers
